# Diversity and community pattern of sulfate-reducing bacteria in piglet gut

**DOI:** 10.1186/s40104-019-0346-5

**Published:** 2019-05-13

**Authors:** Shuwen Ran, Chunlong Mu, Weiyun Zhu

**Affiliations:** 1 Laboratory of Gastrointestinal Microbiology, Jiangsu Key Laboratory of Gastrointestinal Nutrition and Animal Health, College of Animal Science and Technology, Nanjing Agricultural University, No. 6, Tongwei Road, Nanjing, 210095 Jiangsu China; 2 National Center for International Research on Animal Gut Nutrition, Nanjing Agricultural University, Nanjing, 210095 China

**Keywords:** Breeds, Community structure, Piglets, Sulfate-reducing bacteria

## Abstract

**Background:**

Among the gut microbiota, sulfate-reducing bacteria (SRB) is a kind of hydrogen-utilizing functional bacteria that plays an important role in intestinal hydrogen and sulfur metabolism. However, information is lacking regarding diversity and community structure of SRB in the gut of piglets. Middle cecum contents were collected from 6 Yorkshire and 6 Meishan piglets at postnatal days (PND) 14, 28 and 49. Piglets were weaned at PND28. Real-time quantitative PCR was performed to detect the number of SRB in the cecum based on dissimilatory sulfite reductase subunit A (*dsrA*) gene. Prior to real-time PCR, plasmid containing the *dsrA* gene was constructed and used as external standard to create a standard curve, from which the gene copies of *dsrA* were calculated. H_2_S concentration in the cecal contents was measured. Illumina PE250 sequencing of *dsrA* gene was used to investigate SRB diversity in cecum contents.

**Results:**

The qPCR results showed that the number of SRB at PND49 was significantly higher than that at PND28 in Meishan piglets. The concentration of H_2_S has no significant difference between piglet breeds and between different ages. The Illumina sequencing analysis revealed that the Chao1 richness index was significantly higher at PND49 than that at PND14 and PND28 in Yorkshire piglets. Based on *dsrA* gene similarities, Proteobacteria, Actinobacteria, and Firmicutes were identified at the phylum level, and most sequences were classified as Proteobacteria. At the genus level, most of sequences were classified as *Desulfovibrio*. At the species level, *Desulfovibrio intestinalis* was the predominant SRB in the piglet cecum. The relative abundance and the inferred absolute abundance of *Faecalibacterium prausnitzii* at PND49 were significantly higher than that at PND14 in Yorkshire piglets. Pig breeds did not affect the *dsrA* gene copies of SRB, diversity index and community pattern of SRB.

**Conclusions:**

Sulfate-reducing bacteria are widely colonized in the cecum of piglets and *D. intestinalis* is the dominant SRB. The age of piglets, but not the pig breeds affects the diversity and community pattern of SRB.

**Electronic supplementary material:**

The online version of this article (10.1186/s40104-019-0346-5) contains supplementary material, which is available to authorized users.

## Background

Sulfate-reducing bacteria (SRB) are important hydrogen-utilizing bacteria that are colonized in the digestive tract of mammals and in the natural environment. They utilize H_2_, lactate, and acetates as electron donors and sulfate or sulfite as electron acceptors to produce H_2_S. An increase in intestinal H_2_S has been linked to inflammatory bowel disease (IBD) because it causes injury to the intestinal mucosa and inhibits butyrate oxidization [1]. The timely removal of H_2_ from lumens by SRB is indispensable to keep the healthy gut. Therefore, SRB serves as indispensable functional bacteria in gut.

SRBs are not a single bacteria but a group of bacteria which have similar functions, physiology and ecology [2]. To our knowledge, the species within genera *Desulfovibrio*, *Desulfobacter*, *Desulfobulbus* and *Desulfotomaculum* are the most often studied SRB in human and animals, and the genus *Desulfovibrio* is the most abundant (67%~ 91%) in the human gut [3]. However, the diversity and community pattern of SRB in piglets’ gut remain unclear.

The Meishan pig is a domestic Chinese obese breed, while the Yorkshire pig is a commercial lean breed. Earlier studies have found that the intestinal hydrogen metabolism capability of Meishan and Yorkshire piglets differed, as shown by a shift in hydrogenotrophic methanogen colonization [4]. Specifically, the substitution speed of *Methanobrevibacter smithii* for *Methanobrevibacter thaueri/ Methanobrevibacter millerae* was faster in Yorkshire piglets than in Meishan piglets [4]. In addition to methanogens, SRBs are also efficient hydrogenotrophs. Whether Meishan and Yorkshire piglets differ in SRB compositions remains unclear. Therefore, this study hypothesized that a difference in the diversity of SRB exists between Yorkshire and Meishan piglets.

The marker gene encoding dissimilatory sulfite reductase (*dsr*) is widely used to investigate the diversity and quantity of SRB [5]. *Dsr* consists of two subunits: *dsrA* and *dsrB*. *DsrA* is the binding subunit of the *dsr* complex, and *dsrB* is the catalytic subunit [6]. *DsrA* is involved in the energy metabolism of SRB and serves as a reliable genetic marker to study intestinal SRB [7]. Other than *dsrA*, adenosine-5′-phosphosulfate reductase (*apr*) and 16S rRNA have also been widely used to detect SRB.

Current development of next-generation sequencing technology allows for profiling the bacteria composition based on functional genes. However, the quantity of SRB would be overestimated by targeting the *Desulfovibrio* 16S rRNA [8]. A scarcely low abundance of SRB was detected in a piglet gut using 16S rRNA-based Illumina sequencing [9]. Employing the *dsrA*-based Illumina PE250 sequencing, the present study catalogued the previously underrepresented composition of SRB in the piglet gut using cecum contents from two pig breeds. These results dissected the diversity of SRB colonized in piglets’ gut and provided reference for future research on SRB’s interaction with other bacteria and with the host.

## Materials and methods

### Experimental design and sample collection

The animal experiment was carried out at the livestock farm located in Jiangsu Province, China. Animals were managed throughout the study in accordance with requirements for the Experimental Animal Care and Use guidelines of Chinese Science and Technology Committee, 1998. Six Meishan sows and six Yorkshire sow were applied estrus synchronization to ensure they had similar farrowing date. Different sows were fed the same diets. Each litter contains 10-12 piglets. From PND14, all suckling piglets were provided with creep feeding ad libitum and had free access to water. Diets were the same for different piglets. Piglets were weaned at PND28. On the weaning day, the sows were removed from each pen to avoid stress by changing environment. At postnatal days (PNDs) 14, 28 and 49, one piglet from each litter was randomly selected for sampling. After dissecting the abdominal cavity, the middle cecum was ligated and excised from the distal ileum and proximal colon. Cecum contents were collected in sterile tubes and then immediately stored at − 80 °C for further analysis.

### Measurements of sulfide concentrations

The concentration of sulfide (μmol/g wet content) in the cecum contents was measured using a commercially available kit (Cat. No.: A146, Jiancheng Bioengineering Institute, Nanjing, China) following the instruction of manufacture. Hydrogen sulfide were detected by measuring the methylene blue formation reaction from sulfide and N-amino dimethylaniline .

### Bacterial genomic DNA extraction

Total genomic DNA was extracted from 0.3 g cecum contents using the bead-beating and phenol-chloroform extraction methods as previously described [10]. The quantity and quality of the extracted DNA was measured by a NanoDrop 1000 spectrophotometer (Thermo Scientific Inc., Wilmington, DE, USA), and stored at − 80 °C before further analysis.

### Quantitative real-time PCR

Before conducting real-time PCR assay, the PCR amplicon of *dsrA* gene was purified and cloned into pUCm-T vector (Sangon Biotech, Shanghai, China). And the primers for SRB were *dsrA*-F:5´-ACSCACTGGAAGCACGGCGG-3´ and *dsrA*-R:5´-GTGGMRCCGTGCAKRTTGG-3´ [11]. The reconstructed plasmids were transferred into *Escherichia coli* DH5α (TIANGEN, Beijing, China) to obtain plasmid containing *dsrA* gene. Positive clones were selected for enrichment culture by blue-white screening and subsequent plasmid extraction. The plasmid was extracted using a commercially available kit (E.Z.N.A.® Plasmid Mini Kit I, V (capped) Spin: Solarbio Bioengineering Institute, Beijing, China) and measured by a NanoDrop 1000 spectrophotometer (Thermo Scientific Inc., Wilmington, DE, USA). Real-time PCR assay was performed on a QuantStudio™ 7 Flex Real-Time PCR System (Applied Biosystems) with ROX reference dye and SYBR fluorescence dye (TaKaRa Biotechnology, Dalian, China). The PCR amplication was performed as previously described [12]. A 10-fold dilution series of the standard plasmid for the related target was also run with the samples to prepare standard curve. The copy number of each sample was calculated based on the copy number of series of the standard plasmid.

### Illumina PE250 sequencing based on *dsrA* gene

DNA used to perform Illumina sequencing was same as that in the real-time PCR procedure. PCR reactions were performed in triplicate with 20 μL mixture containing 4 μL of 5-fold FastPfu Buffer, 2 μL of 2.5 mmol/L dNTPs, 0.8 μL of each primer (5 μmol/L), 0.4 μL of FastPfu Polymerase, and 10 ng of template DNA. The primers for sequencing were *dsrA*-F:5´-ACSCACTGGAAGCACGGCGG-3´ and *dsrA*-R:5´-GTGGMRCCGTGCAKRTTGG-3´. The cycling parameters were as follows: 95 °C for 2 min, followed by 25 cycles at 95 °C for 30 s, 55 °C for 30 s, and 72 °C for 30 s and a final extension at 72 °C for 5 min. A mixture of amplicons was detected by 2% agarose gel electrophoresis. Purification and quantification were carried out by the AxyPrep DNA Gel Extraction Kit (Axygen Biosciences, Union City, CA, USA) and QuantiFluor™ -ST (Promega, USA), respectively.

Every twenty-four amplicons with different barcodes were mixed equally and the pooled DNA product was used to construct Illumina Pair-End library following Illumina’s genomic DNA library preparation procedure. Then the amplicon library was paired-end sequenced on an Illumina PE250 platform (Shanghai BIOZERON Co, Ltd) according to the standard protocols.

### Bioinformatics analysis

After sequencing, the raw reads were deposited into the NCBI Sequence Read Archive (SRA) database (SRP186735). QIIME (version 1.17) was used to quality-filtered of raw fastq based on following criteria: (1) The 250 bp reads were truncated at any site receiving an average quality score < 20 over a 10-bp sliding window, discarding the truncated reads that were shorter than 50 bp; (2) exact barcode matching, 2 nucleotide mismatch in primer matching, reads containing ambiguous characters were removed; (3) only sequences that overlap longer than 10 bp were assembled according to their overlap sequence. Reads which could not be assembled were discarded.

Operational taxonomic units (OTUs) were clustered with 97% similarity cut off using UPARSE (version 7.1 http://drive5.com/uparse/) and chimeric sequences were identified and removed using UCHIME. The phylogenetic affiliation of each sequence was analyzed using the using FunGene (http://fungene.cme.msu.edu/) with a confidence threshold of 70%. After rarefied based on minimum number of reads obtained in a sample, we calculated the diversity indices, including Chao 1, Coverage index, Shannon index and Simpson index, using the MOTHUR program (http://www.mothur.org). The effects of age, breed and interaction between the two factors on these diversity indices were tested for significance using a two-way ANOVA program. Student’s *t*-test was used to test significance of these diversity indices among different breeds at the same age. One-Way ANOVA was used to test significance of these diversity indices at different age in the same breed. *P*<0.05 represented significant differences. The principal coordinates analysis (PCoA) was also performed via the unweighted/weighted UniFrac distance method by MOTHUR program (http://www.mothur.org) and AMOVA was used to test significance of PCoA at different age and pig breed. Phylogenetic analysis was performed with the MEGA 6 (http://www.megasoftware.net/) [13]. An unrooted phylogenetic tree was constructed using the neighbour-joining method [14].

### Statistical analysis

All results were expressed by mean ± standard error. SPSS 20.0 were used to carry out statistical analysis. The effects of age, breed and interactions between the two factors on the concentration of H_2_S and the *dsrA* gene copies were tested for significance using a two-way ANOVA program. Student’s *t*-test was used to test significance of *dsrA* gene copies and concentration of H_2_S among the different breeds at the same age. One-way ANOVA analysis was used to test significance of *dsrA* gene copies and concentration of H_2_S at different age. *P*<0.05 represented significant differences. Kruskal-Wallis One-Way ANOVA was used to test significance of relative abundance and inferred absolute abundance of specific bacteria at different age and different age. After that false discovery rate analysis was performed to adjust the *P* value and *q* value<0.05 represented significant differences.

## Results

### Effects of breed and age on *dsrA* gene copies and sulfide concentrations

**Fig. 1:**
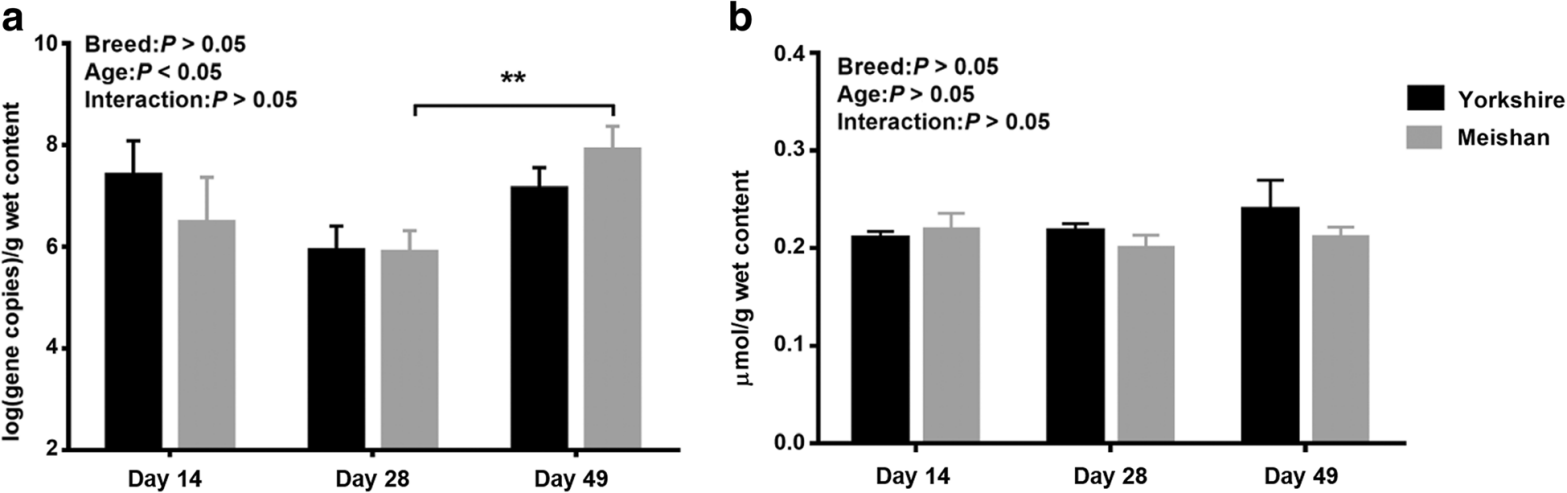
**a** The dsrA gene copies in cecum digests of Yorkshire piglets and Meishan piglets. **b** The concentration of sulfide in cecum contents. *X*-axis represents different age, and *Y*-axis represents bacterial copy number * represents *P* < 0.05, ** represents *P* < 0.01

The *dsrA* gene copies were measured to quantify the total numbers of SRB in the cecum digesta. As shown in Fig. 1a, there existed significant difference of the *dsrA* gene copies at different age (*P* < 0.05). Specifically, in Meishan piglets, the *dsrA* gene copies were higher at PND49 than that at PND28 (*P* < 0.01). However, significant differences were not found in Yorkshire piglets. (Fig. 1b). Breed did not affect the *dsrA* gene copies (Fig. 1a) and sulfide concentrations in the cecum digesta (Fig. 1b) during the studied period.

### Diversity of SRB in the cecum of piglets

**Fig. 2:**
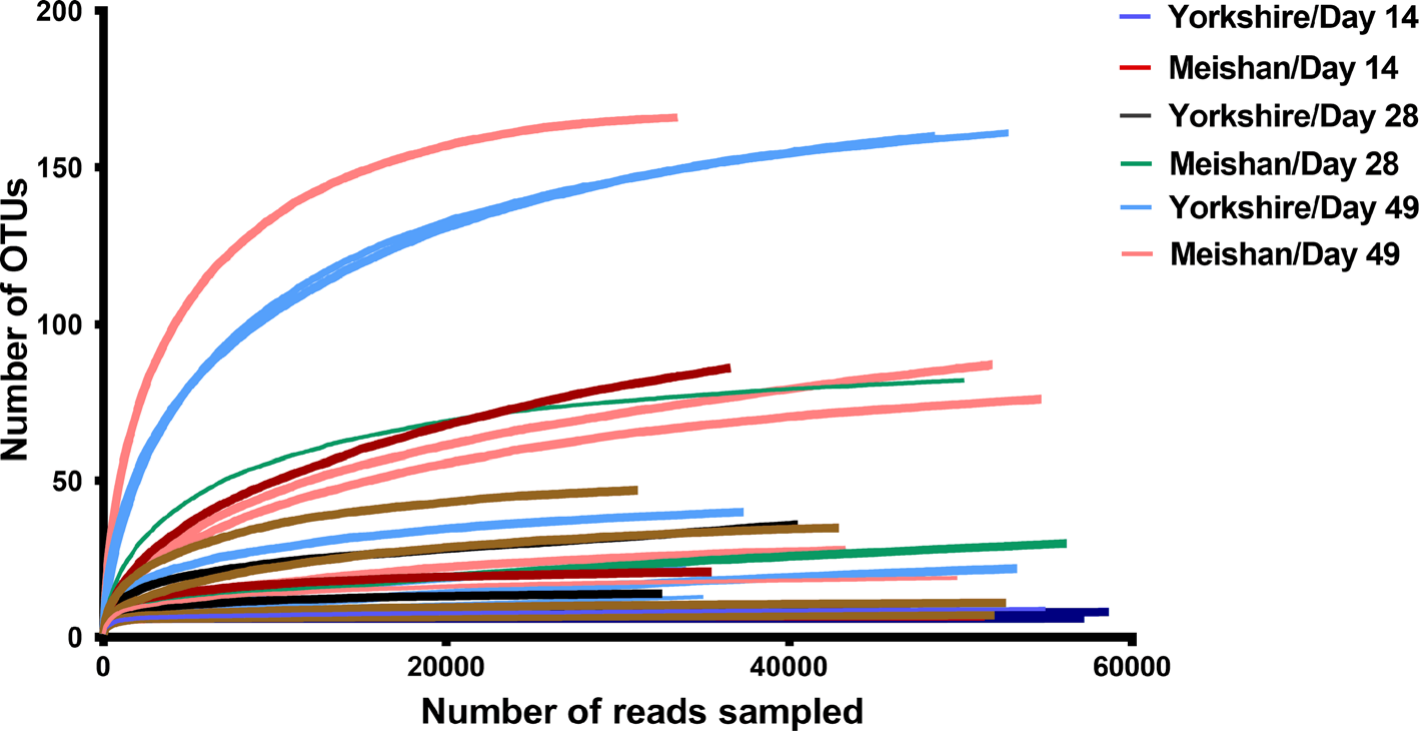
Rarefaction curves comparing the number of reads with the number of phylotypes (OTUs) found in the gene libraries from cecum contents of Meishan and Yorkshire piglets

**Fig. 3:**
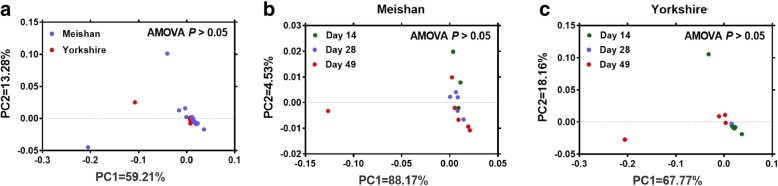
Principal coordinate analysis of SRB using unweighted unifrac dissimilarities between the Meishan piglets and the Yorkshire piglets (**a**), at days 14, 28 and 49 in Meishan piglets (**b**) and at days 14, 28 and 49 in Yorkshire piglets (**c**)

Since the real-time PCR only showed the total *dsrA* gene copies but not the detail bacteria composition, we further used *dsrA*-based Illumina sequencing to analyze the diversity of SRB. Across all samples, 1,441,175 trimmed sequences were obtained. 130 OTUs were identified after removing OTUs that contained less than 3 sequences. The rarefaction curves tended to approach the saturation plateau (Fig. 2). As a marker of the sequencing accuracy, coverage index was close to 100% for all samples (Table 1), indicating that the current Illumina sequencing covers most of the SRB sequences of samples. There was no significant difference at different days on Simpson index and Shannon index, which were the most sensitive markers to changes of the most abundant species and the rare species, respectively. The higher Shannon index and the lower Simpson index all indicated higher bacteria diversity. The Chao index was usually considered as a marker of bacteria richness, which was used to estimate the number of OTU in community, and Table 1 revealed that Chao index significantly increased with age in Yorkshire piglets (*P* < 0.05), however, the difference was not detected in Meishan piglets. Breed did not affect the Simpson index, Shannon index and Chao index (Table 1). Principal coordinate analysis (PCoA) indicated that there was no significant difference on the community pattern of SRB at different days and different breeds (Fig. 3 and Additional file 1: Figure S1).

**Table 1:** Phylotype coverage and diversity estimation of the SRB in Yorkshire and Meishan piglets

Breed	Age, d	Chao	Shannon	Simpson	Coverage
Meishan	14	52.88	0.37	1.18	0.99963233
	28	21.82	0.31	1.15	0.99988833
	49	40.27	0.41	1.20	0.99973383
	14	12.56	0.32	1.17	0.999954
Yokshire	28	7.18	0.31	1.16	0.9999802
	49	50.34	0.55	1.31	0.9997105
SEM		29.79	0.213	0.151	0.0002
Effect (*P* value)	Breed	0.131	0.684	0.513	0.055
	Age	0.032	0.140	0.263	0.027
	Breed×Age	0.128	0.605	0.650	0.122

### The phylogenetic community analysis of SRB

**Fig. 4:**
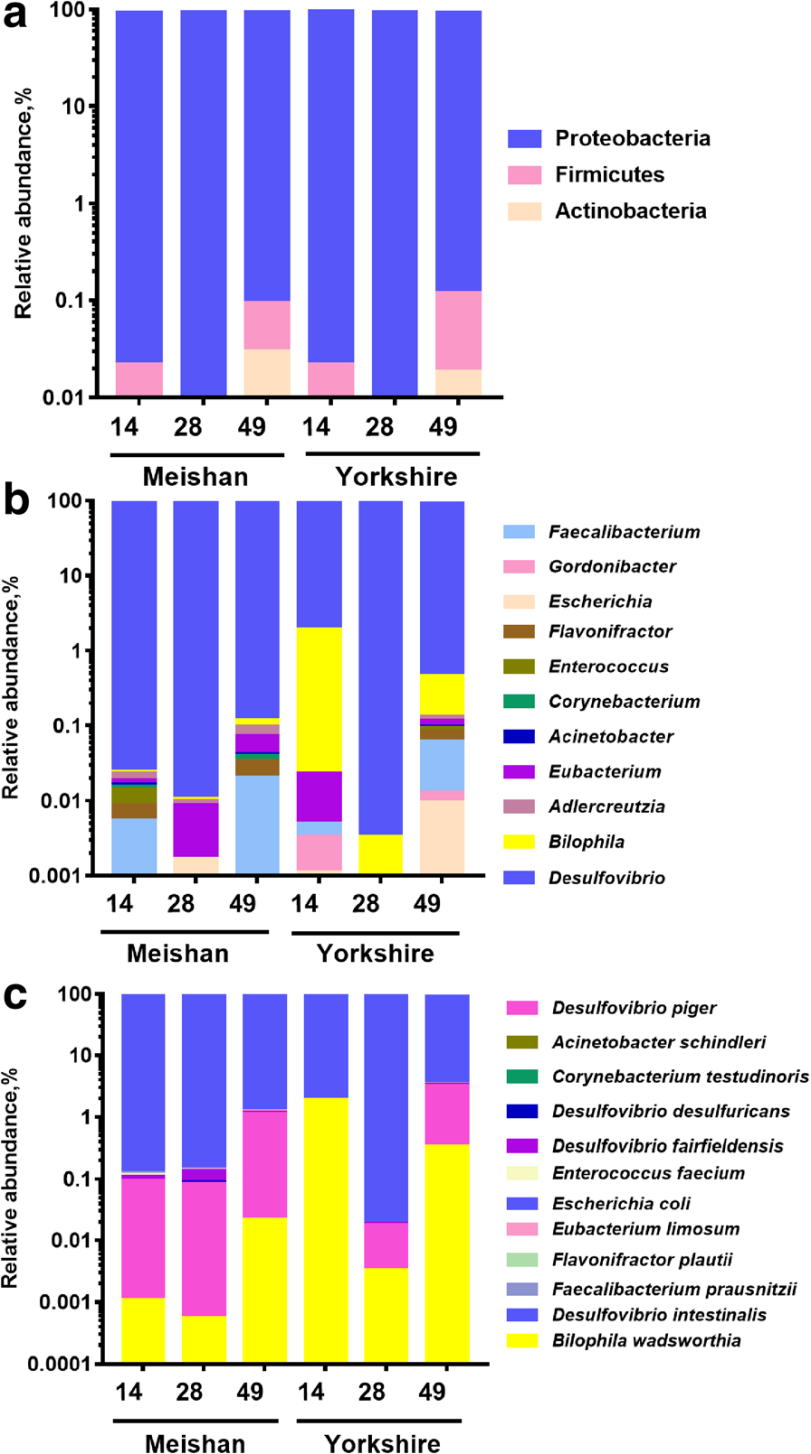
The community structures of SRB in the cecum contents of piglets at the phylum levels (**a**), genus levels (**b**) and species levels (**c**). Unclassified bacteria were not presented in the figure

After analyzing the diversity of total SRB in piglets’ gut, we further analyzed the community pattern of SRB. We found that at the phylum level, most of the sequences were classified as Actinobacteria, Firmicutes and Proteobacteria, and majority of sequences were classified as Proteobacteria (Fig. 4a). At the genus level, we detected 11 genera in the two pig breeds, and most species belonging to *Desulfovibrio* (Fig. 4b). At the species level, we were surprised to find that some rare bacteria also have *dsrA* gene, for example *Faecalibacterium prausnitzii*, *Eubacterium limosum*, *Enterococcus faecium*. *Desulfovibrio intestinalis* was the predominant SRB in Meishan and Yorkshire piglets. Additionally, we found that *Bilophila wadsworthia* was the second largest SRB in Yorkshire piglets and *Desulfovibrio piger* was the second abundant SRB in Meishan piglets (Fig. 4c)*.*

### Diversity of SRB at different age and in different breeds

Relative abundance and absolute abundance were widely used to investigate microbiota composition. To detect the quantity of specific bacteria at different days, we multiplied the gene copies of *dsrA* which was detected by real-time PCR and proportions of each bacterium which were detected by sequencing to calculate the inferred absolute abundance of each bacterium. Then, we found that age affected the relative abundance and the inferred absolute abundance of SRB.

**Fig. 5:**
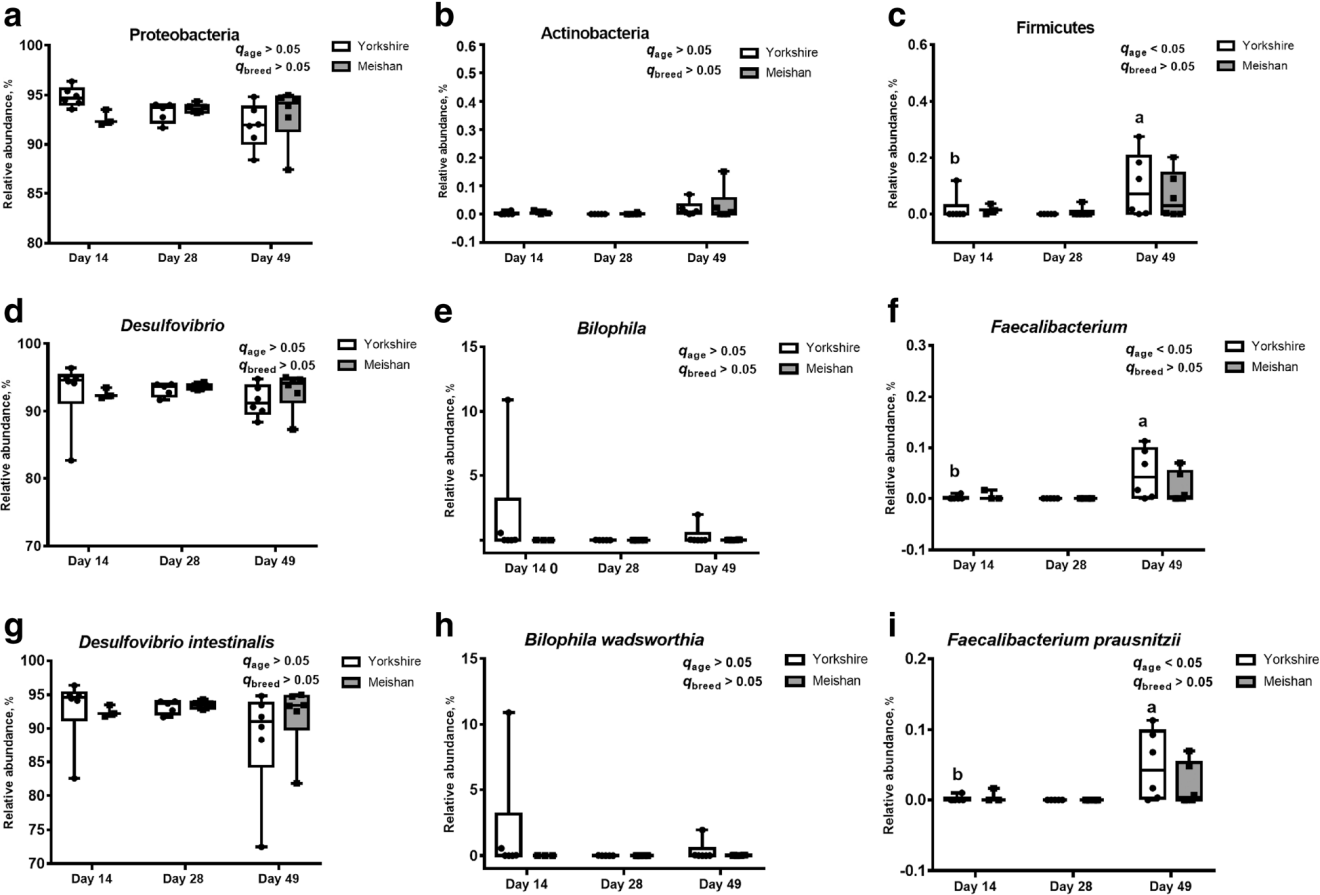
The relative abundance of SRB at the phylum levels (**a-c**), genus levels (**d-f**) and species levels (**g-i**). Different letters represent difference at different age in the same breed, *q* < 0.05

As shown in Fig. 5c, at the phylum level, Firmicutes at PND49 increased significantly by relative abundance on Yorkshire piglets (*q* < 0.05). At the genus level, *Faecalibacterium* was significantly higher at PND49 than that at PND14 by relative abundance in Yorkshire piglets (*q* < 0.05, Fig. 5f). Figure 5i indicated that at the species level, the relative abundance of *F. prausnitzii* significantly increased at PND49 comparing to that at PND14 (*q* < 0.05) in Yorkshire piglets. Pig breeds did not affect relative abundance of these bacteria.

**Fig. 6:**
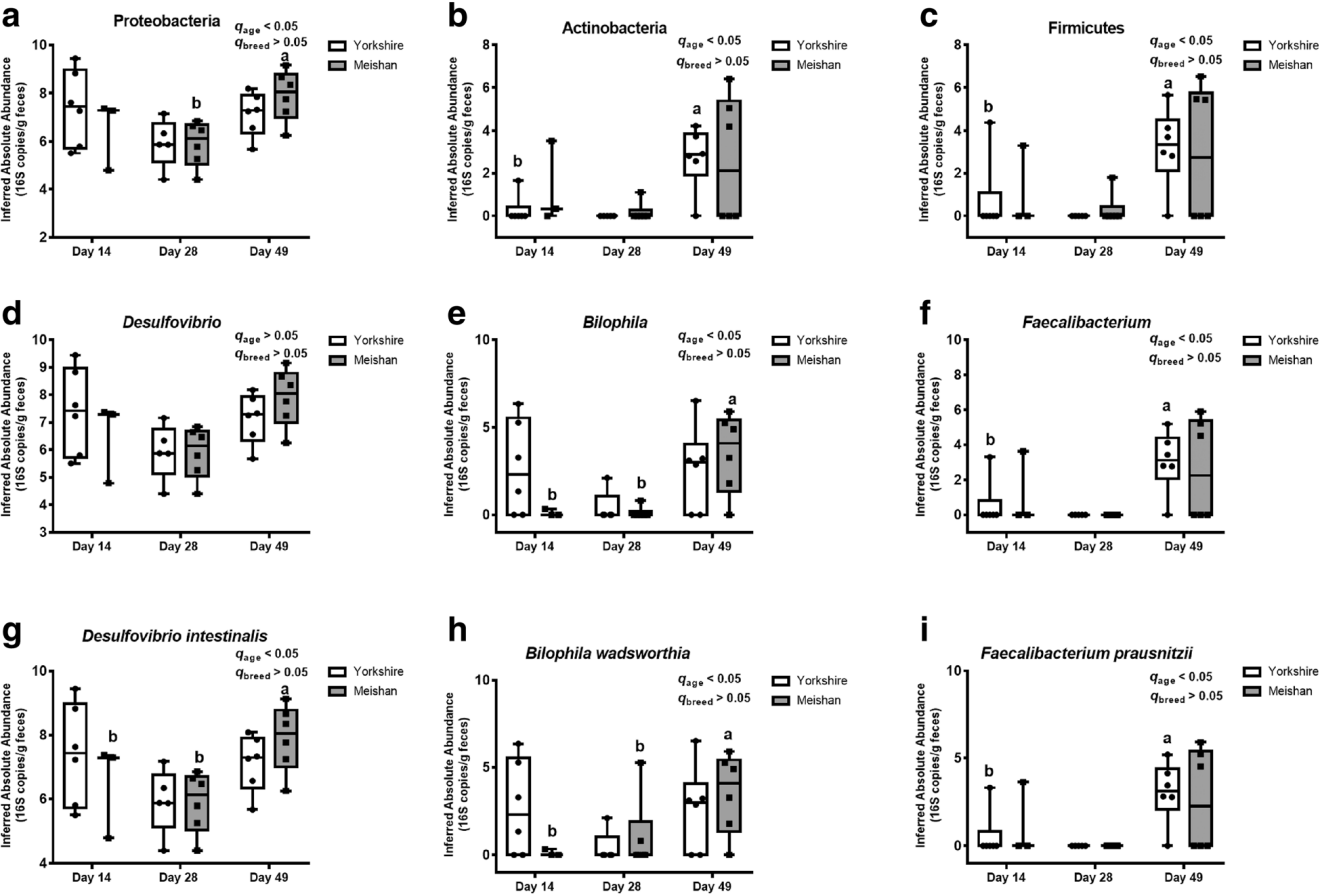
The inferred absolute abundance of SRB at the phylum levels (**a**-**c**), genus levels (**d**-**f**) and species levels (**g**-**i**). Different letters represent difference at different age in the same breed, *q* < 0.05

**Fig. 7:**
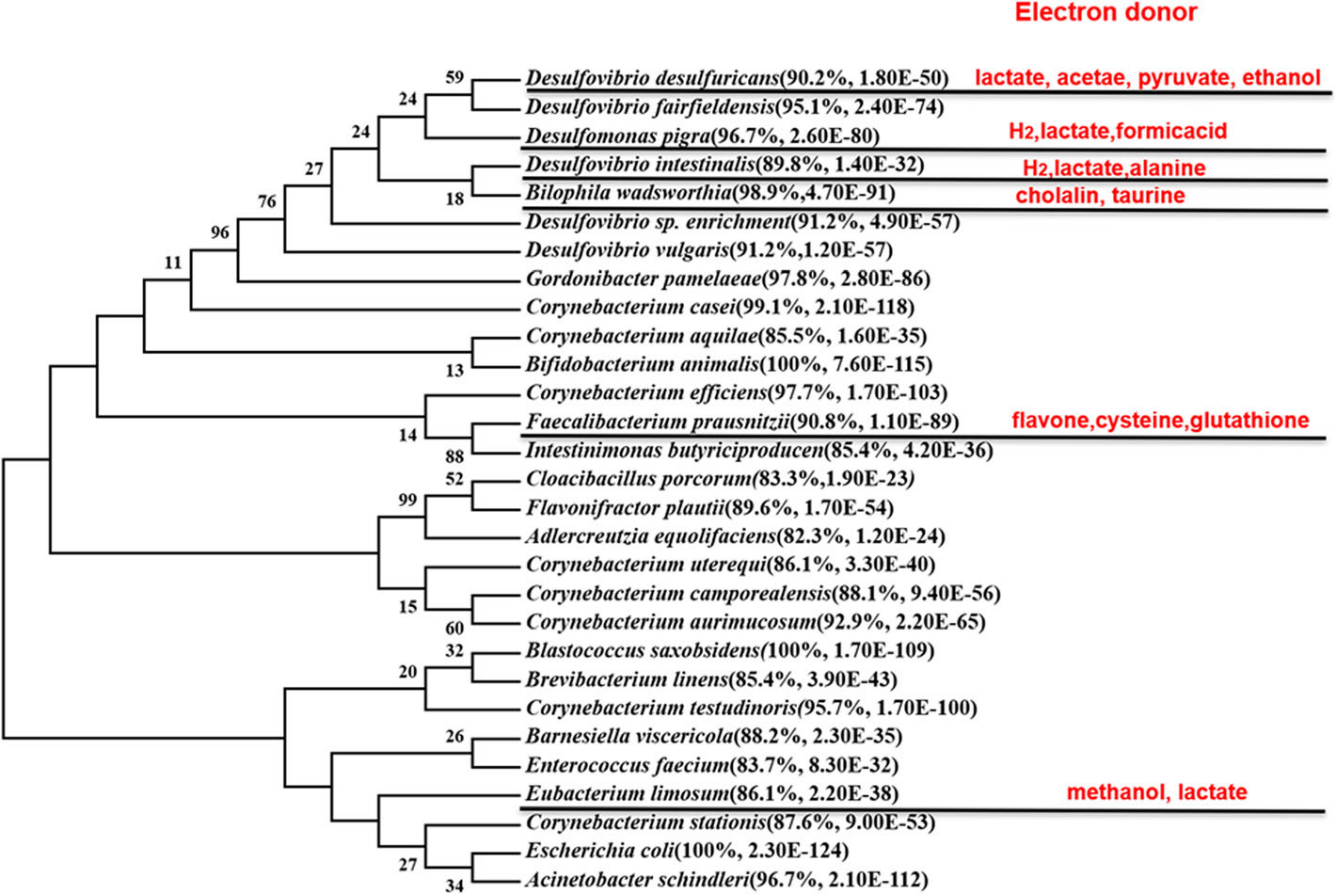
Phylogeny of SRB constructed by MEGA 6. Electron donors were listed according to previous studies. The similarity and E-value against the known sequences from NCBI blast were shown

In addition to the relative abundance of SRB, age also affected the inferred absolute abundance of SRB. At the phylum level, Firmicutes and Actinobacteria significantly increased (*q* < 0.05) at PND49 comparing to PND14 by inferred absolute abundance in Yorkshire piglets, and the inferred absolute abundance of Proteobacteria at PND49 was significantly higher than that at PND28 (*q* < 0.05) in Meishan piglets (Fig. 6a, b and c). Figure 6e and f showed that the inferred absolute abundance of *Bilophila* at PND49 was significantly higher than that at PND28 and PND 14 on Meishan piglets (*q* < 0.05), and the inferred absolute abundance of *Faecalibacterium* at PND49 was significantly higher than that at PND14 in Yorkshire piglets (*q* < 0.05). At the species level, *B. wadsworthia* and *D. intestinalis* significantly (*q* < 0.05) increased at PND49 comparing to PND14 and PND28 by inferred absolute abundance in Meishan piglets, and the inferred absolute abundance of *F. prausnitzii* significantly (*q* < 0.05) increased at PND49 comparing to PND14 in Yorkshire piglets (Fig. 6g, h and i). It was mentioned that *F. prausnitzii* in Yorkshire piglets changed significantly both in the relative abundance and the inferred absolute abundance. After excluding OTUs that were not classified, we constructed the phylogenetic tree of SRB based on *dsrA* gene, which contains 29 species that may colonize in the piglets’ gut (Fig. 7). Based on previous culture-based studies, the substrates that specific bacteria can utilized were shown in the phylogenetic tree. Culture-based studies supporting substrate utilization are essential to make the inference. Here we try to provide clues for potential substrate utilization of those SRB identified in the present study.

Interestingly, different SBB could use different substrates as electron donor to produce H_2_S. *Desulfovibrio desulfuricans* could utilize lactate, acetate, pyruvate, ethanol to produce H_2_S [15]. *D. piger* use H_2_, lactate, formic acid as electron donor [16]. *D. intestinalis* use lactate, H_2_, alanine [17]. Unlike other SRB, *B. wadsworthia* uses cholalin and taurine to produce H_2_S [18]. Some Sulphur-containing amino acid could also be used as electron donor. Flavone, cysteine and glutathione could be used by *F. prausnitzii* [19]. *E. limosum* also has the ability to produce H_2_S by methanol and lactate [20].

## Discussion

SRBs are a major group of hydrogenotrophic bacteria in the gut that exert important roles on intestinal hydrogen removal and fermentation. A comprehensive illustration of SRB composition is a prerequisite for further study on their interaction with the host. The present study combined real-time PCR and Illumina sequencing analysis to investigate the diversity of SRB in the gut of piglets. The study found that *Desulfovibrio intestinalis* was the predominant SRB in the piglet gut. Other species, such as *B. wadsworthia* and *F. prausnitzii*, were also identified using *dsrA*-based sequencing. Furthermore, the age of the piglets was found to affect SRB diversity. No difference was found in the quantity of SRB, the diversity index, or the community pattern between the Meishan piglets and the Yorkshire piglets.

### Illumina PE250 sequencing: a sensitive method to investigate diversity and community structure of SRB

Current development in Illumina sequencing enabled the researchers to profile bacteria composition based on sequencing of function genes. Employing the *dsrA*-based sequencing, the present study catalogued the previously underrepresented composition of SRB in the piglet gut.

Both culture-dependent and independent methods have been used to study SRB composition. Kushkevych isolated 20 pure cultures belonging to *Desulfovibrio* sp. and *Desulfomicrobium* sp. from the human large intestine and found that these bacteria could form round and black colonies on solid media [21]. Shukla and colleagues isolated *Desulfovibrio desulfuricans* from blood samples of dogs with disease using anaerobic chocolate agar plates [15]. Culture-independent methods, such as Illumina sequencing and polymerase chain reaction-denaturing gradient electrophoresis (PCR-DGGE) have also been widely applied in microbiome studies. PCR-DGGE analysis of the *aprA* gene found that SRB are common bacteria in the human gut and that each individual harbors 1 to 5 SRB phylotypes. Of these, *D. desulfuricans*, *D. intestinalis*, and *Desulfovibrio piger* were the dominant species [22].

In the present study, *dsrA*-based sequencing found that an average of 45,037 sequences were classified as SRB per sample. In addition to the common SRB, *Desulfovibrio*, *F. prausnitzii*, *Adlercreutzia equolifaciens*, *Eubacterium limosum,* and *Enterococcus faecium* were also found to have the *dsrA* gene. Therefore, Illumina PE250 sequencing based on the *dsrA* gene was shown to be a sensitive method to study the diversity and community structure of SRB in piglet guts***.***

### *D. intestinalis*: the predominant SRB in the cecum of piglets

*D. intestinalis* was found to be the most abundant SRB in the piglet gut regardless of age. In addition, *D. intestinalis* was also found in termite [17] and human [23]. *D. intestinalis* could use sulfate, sulfite, and thiosulfate as electron acceptors and formate, pyruvate, and lactate as electron donors to produce H_2_S [17]. Although the function of *D. intestinalis* in the porcine gut remains unclear, its widespread existence in animals and humans indicated that the bacterium is necessary to maintain homeostasis functions for the host.

The present results also revealed that in Yorkshire piglets, *B. wadsworthia* was the second dominant SRB, which was similar to the composition found in the human gut [24]. However, in Meishan piglets, *D. piger* was the second dominant SRB in the cecum. Interestingly, the two bacteria may be potentially opportunistic pathogens. *B. wadsworthia* can induce hepatic abscesses [25] and perforated appendicitis [23]. *D. piger* was deemed to be involved in the incidence of IBD [26]. Colonization of *D. piger* in gnotobiotic mice increased the production of H_2_S and decreased the expression of claudin-4 in the colonic mucosa of mice [16]. Various factors could influence the gastrointestinal bacterial flora, including genetic, environment, diet, and health condition factors. It might be speculated that the specific SRB is colonized in specific animal species to adapt environment.

### Age of pigs: a predominant influencer on the diversity of SRB

In the present study, the gene copies of SRB increased with age in Meishan piglets. A similar phenomenon has also been found in humans and elderly people showed more intestinal SRB than teenagers [27]. This may be due to the change of diet or endogenous substrate after weaning, such as chondroitin sulphate and mucins [28].

Based on the gene copies of *dsrA*, the inferred absolute quantity of each bacterium was calculated by multiplying the relative abundance from the Illumina sequencing analysis with *dsrA* gene copies. *F. prausnitzii* in the Yorkshire piglets were found to change with age, considering the relative abundance and the inferred absolute quantity. *F. prausnitzii* are regarded as a healthy biomarker of the human gut because they produce butyrate, which supplies energy to intestinal parietal cells and promotes intestinal health [29]. *F. prausnitzii* can cross-feed with *Bacteroides thetaiotaomicron* to modulate gut physiology [30]. *F. prausnitzii* supernatant can inhibit NF-κB signaling of Caco2 cells and ameliorate 2,4,6-trinitrobenzene sulfonate sulphonic acid-induced colitis in mice by increasing IL-10 expressions [31]. We speculated that the significant augment of *F. prausnitzii* at postnatal day 49 may have been the result of adaptation to changes in intestinal environment and diets. In the present study, the piglets were weaned at 28 days, and commercial food containing a total nutrient was supplied to the piglets afterward. *F. prausnitzii* can use carbohydrates to produce short-chain fatty acids to improve intestine growth*. F. prausnitzii* may be also involved in protecting the gut against oxidative stress. Additionally, *F. prausnitzii* can inhibit inflammation and oxidative stress of colonic epithelial cells in vitro [32]. The increase of *F. prausnitzii* at day 49 may increase the anti-oxidative function in gut. The decrease of *F. prausnitzii* at day 28 may result from weaning stress, because the bacteria community composition changed significantly around weaning, and some pathogenic bacteria increased while beneficial bacteria decreased [33]*.*

*B. wadsworthia* was also an important SRB that has been widely recognized as a potential pathogen. The present study indicated that it existed in the gut of piglets. The inferred absolute abundance of *B. wadsworthia* increased significantly with age (from 0.11% at day 14 to 2.18% at day 49). Using the PCR method, a previous study also found that *B. wadsworthia* widely existed in the feces of pigs, but no obvious damages of the intestinal tract were observed [34]*. B. wadsworthia* has been reported to use pyruvate and taurine to produce alanine, acetate, and ammonia [35]. Additionally, taurocholic acid can stimulate the growth of *B. wadsworthia* [18]. Therefore, an increase in taurine metabolism and available substrate in the gut may boost the increase of *B. wadsworthia* with piglet growth. Determining whether *B. wadsworthia* has any beneficial functions in the gut requires further study.

Pig breed did not affect the diversity of SRB. Similarly, breed did not affect the abundance of hydrogen-utilizing archaea in the piglet gut. Su and colleagues found that the diversity of the methanogenic community was also influenced mainly by the age of piglets but not the breeds [4]. However, breed was found to affect gut SRB composition in non-human primate species using *dsrB*-targeted analysis [36]. This difference was most likely due to the difference in animal species and the target gene used in different study.

In addition to the role of SRB in metabolism, SRB is also implicated in IBD pathogenesis. Figliuolo et al. found that administrating *Desulfovibrio indonesiensis* or an SRB mixture obtained from colitis patients to germ-free mice led to the activation of T cells and upregulated IL-6, IL-17, IFN-γ, and TNF-α in mesenteric lymph node cells [37]. An increase in *D. desulfuricans* and *Desulfovibrio vulgaris* was found in the colon of patients with ulcerative colitis (UC) [38]. In addition to *Desulfovibrio*, other SRB, such as *B. wadsworthia*, also led to inflammation*. B. wadsworthia* treatment reduced body weight and increased blood amyloid A and IL-6 in C57BL/6 mice [39]*.* An increase in *B. wadsworthia* mediated the colitis induced by a high saturated fat diet in IL-10-deficient mice [18]*.* These findings implicated the potential regulation of SRB in IBD. Inflammation caused by SRB may result from metabolites of H_2_S, which have been found to damage intestinal epithelial cells by inhibiting butyrate oxidation [40, 41]. Lipopolysaccharide of SRB may also contribute to inflammation [42]*.* Molybdate has been reported to inhibit the sulfate reduction by affecting transportation of sulphate to bacterial cells and the formation of energy, thereby inhibiting the growth of SRB [43]. Other than molybdate, 5-aminosalicylic acid-containing drugs have also been used to treat ulcerative colitis resulting from SRB, because the substances inhibited sulfate reduction and metabolism of sulfur-containing amino acids [41]. These findings provide references for future research on the role of SRB in gut health by using specific compounds targeting SRB.

### Further prospects concerning cross-talk between SRB and other bacteria

The balance between hydrogenotrophic microbes (SRB, methanogens) and hydrogenogenic microbes (*Clostridium*, *Bacteroides*, *Escherichia*) is crucial to maintain hindgut fermentation. SRBs, such as *Desulfovibrio*, utilize H_2_ produced from *Clostridium* and *Bacteroides*. Meanwhile, the methanogen *Methanobrevibacter* has a stronger ability to compete with *Desulfovibrio* for utilizing H_2_. Using a similar experiment setting, Su et al. found that *Methanobrevibacter smithii*-related OTU increased with age and became dominant methanogens from postnatal day 1 to 14 [4]. Bian et al. found that Bacteroidetes (H_2_-producing bacteria) and *Blautia* (H_2_-utilizing acetogens) increased from postnatal day 1 to day 49, while *Escherichia* decreased with age [44]. In the present study, the dominant SRB *D. intestinalis* also increased with age. Overall, these results indicated an increased intestinal fermentation relating to an increase in hydrogenotrophic microbes with age in piglets.

The cross-talk between SRB and other commensal intestinal bacteria can be studied further*. Bacteroides fragilis* and *B. thetaiotaomicron* could release sulfate from mucus to supply source for SRB [45]. Federico and colleagues also found that *B. thetaiotaomicron* boosted growth of *D. piger* through provision of free sulfate, and *Collinsella aerofaciens* could cross-feed with *D. piger* by their metabolite [16]*. D. desulfuricans* could influence the metabolic activity of saccharolytic and amino acid-fermenting bacteria [46]*.* Cross-feeding between different bacteria is important to maintain intestinal homeostasis. Future investigation about cross-feeding between SRB and other bacteria is needed to better understand sulfur metabolism in the gut.

## Conclusion

The present study found that diverse SRBs colonized in the gut of piglets. *D. intestinalis* was the predominant SRB. The age of piglets affected the gene copies of *dsrA*, diversity index, the relative abundance and the inferred absolute abundance of *F. prausnitzii* in Yorkshire piglets, but there was no difference on the diversity and community pattern of SRB between Meishan and Yorkshire piglets. These findings gain more insight into bacterial structure in porcine gut and provide reference for future cultivation-based functional studies.

## Additional file

Additional file 1: **Figure S1.** Principal coordinate analysis of SRB using weighted unifrac dissimilarities between the Meishan piglets and the Yorkshire piglets (A), at days 14, 28 and 49 in Meishan piglets (B) and at days 14, 28 and 49 in Yorkshire piglets (C). (TIF 55 kb)

## Abbreviations

*dsrA*: Dissimilatory sulfite reductase subunit A
OTUs: Operational taxonomic units
PCoA: Principal coordinates analysis (PCoA)
PND: Postnatal days
SRB: Sulfate-reducing bacteria

## References

[1] Deplancke B, Hristova KR, Oakley HA, McCracken VJ, Aminov R, Mackie RI (2000). Molecular ecological analysis of the succession and diversity of sulfate-reducing bacteria in the mouse gastrointestinal tract. Appl Environ Microbiol.

[2] Castro HF, Williams NH, Ogram A (2000). Phylogeny of sulfate-reducing bacteria. FEMS Microbiol Ecol.

[3] Kushkevych IV (2016). Dissimilatory sulfate reduction in the intestinal sulfate-reducing bacteria. Biol Stud..

[4] Su Y, Bian G, Zhu Z, Smidt H, Zhu W (2014). Early methanogenic colonisation in the faeces of Meishan and Yorkshire piglets as determined by pyrosequencing analysis. Archaea..

[5] Gawel LJ, Ng T, Odom JM, Ebersole RC. Sulfate-reducing bacteria determination and control. US Patent. 1991:4999286.

[6] Ritz NL, Burnett BJ, Setty P, Reinhart KM, Wilson MR, Alcock J (2016). Sulfate-reducing bacteria impairs working memory in mice. Physiol Behav.

[7] Wagner M, Loy A, Klein M, Lee N, Ramsing NB, Stahl DA (2005). Functional marker genes for identification of sulfate-reducing prokaryotes. Methods Enzymol.

[8] Christophersen CT, Morrison M, Conlon MA (2011). Overestimation of the abundance of sulfate-reducing bacteria in human feces by quantitative PCR targeting the *Desulfovibrio* 16S rRNA gene. Appl Environ Microbiol.

[9] Mu C, Yang Y, Su Y, Zoetendal EG, Zhu W (2017). Differences in microbiota membership along the gastrointestinal tract of piglets and their differential alterations following an early-life antibiotic intervention. Front Microbiol.

[10] Su Y, Yao W, Perez-Gutierrez ON, Smidt H, Zhu WY (2008). 16S ribosomal RNA-based methods to monitor changes in the hindgut bacterial community of piglets after oral administration of *Lactobacillus sobrius* S1. Anaerobe..

[11] Spence C, Whitehead T, Cotta M (2008). Development and comparison of SYBR green quantitative real-time PCR assays for detection and enumeration of sulfate-reducing bacteria in stored swine manure. J Appl Microbiol.

[12] Yang YX, Mu CL, Luo Z, Zhu WY (2016). Bromochloromethane, a methane analogue, affects the microbiota and metabolic profiles of the rat gastrointestinal tract. Appl Environ Microbiol.

[13] Lozupone C, Knight R (2005). UniFrac: a new phylogenetic method for comparing microbial communities. Appl Environ Microbiol.

[14] Saitou N, Nei M (1987). The neighbor-joining method: a new method for reconstructing phylogenetic tree. Mol Biol Evol.

[15] Shukla SK, Reed KD (2000). *Desulfovibrio desulfuricans* bacteremia in a dog. J Clin Microbiol.

[16] Rey FE, Gonzalez MD, Cheng J, Wu M, Ahern PP, Gordon JI (2013). Metabolic niche of a prominent sulfate-reducing human gut bacterium. Proc Natl Acad Sci U S A.

[17] Fröhlich J, Sass H, Babenzien HD, Kuhnigk T, Varma A, Saxena S (1999). Isolation of *Desulfovibrio intestinalis sp. nov*, from the hindgut of the lower termite *Mastotermes* darwiniensis. Can J Microbiol.

[18] Devkota S, Wang Y, Musch MW, Leone V, Fehlnerpeach H, Nadimpalli A (2012). Dietary-fat-induced taurocholic acid promotes pathobiont expansion and colitis in IL10^−/−^ mice. Nature..

[19] Miquel S, Martín R, Rossi O, Bermúdezhumarán LG, Chatel JM, Sokol H (2013). *Faecalibacterium prausnitzii* and human intestinal health. Curr Opin Microbiol.

[20] Pacaud S, Loubiere P, Goma G (1985). Methanol metabolism by *Eubacterium limosum* B2: effects of pH and carbon dioxide on growth and organic acid production. Curr Microbiol.

[21] Kushkevych IV (2013). Identification of sulfate-reducing bacteria of human large intestine. Biol Stud.

[22] Liu CL, Yin XC, Long WM, Fei N, Zhao LP, Pang XY (2013). Development of a group-specific PCR-based DGGE for analyzing sulfate-reducing bacteria in human gut. Chin J Micro.

[23] Summanen PH, Jousimies-Somer H, Manley S, Bruckner D, Marina M (1995). *Bilophila wadsworthia* isolates from clinical specimens. Clin Infect Dis.

[24] Scanlan PD, Shanahan F, Marchesi JR (2009). Culture-independent analysis of *Desulfovibrios* in the human distal colon of healthy, colorectal cancer and polypectomized individuals. FEMS Microbiol Ecol.

[25] Kasten MJ, Rosenblatt JE, Gustafson DR (1992). *Bilophila wadsworthia* bacteremia in two patients with hepatic abscesses. J Clin Microbiol.

[26] Loubinoux J, Bronowicki JP, Pereira IA, Mougenel JL, Faou AE (2002). Sulfate-reducing bacteria in human feces and their association with inflammatory bowel diseases. FEMS Microbiol Ecol.

[27] Fite A, Macfarlane GT, Cummings JH, Hopkins MJ, Kong SC, Furrie E (2004). Identification and quantitation of mucosal and faecal *Desulfovibrios* using real time polymerase chain reaction. Gut..

[28] Macfarlane George T., Gibson Glenn R. (1997). Carbohydrate Fermentation, Energy Transduction and Gas Metabolism in the Human Large Intestine. Gastrointestinal Microbiology.

[29] Flint HJ, Scott KP, Duncan SH, Louis P, Forano E (2012). Microbial degradation of complex carbohydrates in the gut. Gut Microbes.

[30] Wrzosek L, Miquel S, Noordine ML, Bouet S, Chevalier-Curt MJ, Robert V (2013). *Bacteroides thetaiotaomicron* and *Faecalibacterium prausnitzii* influence the production of mucus glycans and the development of goblet cells in the colonic epithelium of a gnotobiotic model rodent. BMC Biol.

[31] Sokol H, Pigneur B, Watterlot L, Lakhdari O, Bermúdezhumarán LG, Gratadoux JJ (2008). *Faecalibacterium prausnitzii* is an anti-inflammatory commensal bacterium identified by gut microbiota analysis of Crohn disease patients. Proc Natl Acad Sci U S A.

[32] Sadabad MS, Von Martels JZ, Khan MT, Blokzijl T, Paglia G, Dijkstra G (2015). A simple coculture system shows mutualism between anaerobic *Faecalibacteria* and epithelial Caco-2 cells. Sci Rep.

[33] Su Y, Yao W, Perez-Gutierrez ON, Smidt H, Zhu WY (2008). Changes in abundance of *Lactobacillus spp.* and *Streptococcus suis* in the stomach, jejunum and ileum of piglets after weaning. FEMS Microbiol Ecol.

[34] Mcorist AL, Warhurst M, Mcorist S, Bird AR (2001). Colonic infection by *Bilophila wadsworthia* in pigs. J Clin Microbiol.

[35] Laue H, Denger K, Cook AM (1997). Taurine reduction in anaerobic respiration of *Bilophila wadsworthia* RZATAU. Appl Environ Microbiol.

[36] Nakamura N, Leigh SR, Mackie RI, Gaskins HR (2009). Microbial community analysis of rectal methanogens and sulfate reducing bacteria in two non-human primate species. J Med Primatol.

[37] Figliuolo VR, Dos Santos LM, Abalo A, Nanini H, Santos A (2017). Sulfate-reducing bacteria stimulate gut immune responses and contribute to inflammation in experimental colitis. Life Sci.

[38] Fiachra R, Docherty NG, Madeline M (2010). T Brendan M, J Calvin C, O'connell PR. Bacterial colonization of colonic crypt mucous gel and disease activity in ulcerative colitis. Ann Surg.

[39] Zhou F, Long W, Hao B, Ding D, Ma X, Zhao L (2017). A human stool-derived *Bilophila wadsworthia* strain caused systemic inflammation in specific-pathogen-free mice. Gut Pathog.

[40] Li L, Moore PK (2008). Putative biological roles of hydrogen sulfide in health and disease: a breath of not so fresh air?. Trends Pharmacol Sci.

[41] Pitcher MC, Beatty ER, Cummings JH (2000). The contribution of sulphate reducing bacteria and 5-aminosalicylic acid to faecal sulphide in patients with ulcerative colitis. Gut..

[42] Weglarz L, Parfiniewicz B, Mertas A, Kondera-Anasz Z, Jaworska-Kik M, Dzierżewicz Z (2006). Effect of endotoxins isolated from *Desulfovibrio desulfuricans* soil and intestinal strain on the secretion of TNF-α by human mononuclear ce*lls*. Polish J Environ Stud.

[43] Newport PJ, Nedwell DB (2010). The mechanisms of inhibition of *Desulfovibrio* and *Desulfotomaculum* by selenate and molybdate. J Appl Microbiol.

[44] Bian G, Ma S, Zhu Z, Su Y, Zoetendal EG, Mackie R (2016). Age, introduction of solid feed and weaning are more important determinants of gut bacterial succession in piglets than breed and nursing mother as revealed by a reciprocal cross-fostering model. Environ Microbiol.

[45] Tsai HH, Sunderland D, Gibson GR, Hart CA, Rhodes JM (1992). A novel mucin sulphatase from human faeces: its identification, purification and characterization. Clin Sci (Lond).

[46] Newton DF, Cummings JH, Macfarlane S, Macfarlane GT (1998). Growth of a human intestinal *Desulfovibrio desulfuricans* in continuous cultures containing defined populations of saccharolytic and amino acid fermenting bacteria. J Appl Microbiol.

